# Virtual Reality as a New Approach for Risk Taking Assessment

**DOI:** 10.3389/fpsyg.2018.02532

**Published:** 2018-12-12

**Authors:** Carla de-Juan-Ripoll, José L. Soler-Domínguez, Jaime Guixeres, Manuel Contero, Noemi Álvarez Gutiérrez, Mariano Alcañiz

**Affiliations:** Instituto de Investigación e Innovación en Bioingeniería (i3B), Universitat Politècnica de València, Valencia, Spain

**Keywords:** virtual reality, risk taking, occupational risks, risk attitude, risk perception, stealth assessment, psychophysiological assessment, embodiment

## Abstract

Understanding how people behave when facing hazardous situations, how intrinsic and extrinsic factors influence the risk taking (RT) decision making process and to what extent it is possible to modify their reactions externally, are questions that have long interested academics and society in general. In the spheres, among others, of Occupational Safety and Health (OSH), the military, finance and sociology, this topic has multidisciplinary implications because we all constantly face RT situations. Researchers have hitherto assessed RT profiles by conducting questionnaires prior to and after the presentation of stimuli; however, this can lead to the production of biased, non-realistic, RT profiles. This is due to the reflexive nature of choosing an answer in a questionnaire, which is remote from the reactive, emotional and impulsive decision making processes inherent to real, risky situations. One way to address this question is to exploit VR capabilities to generate immersive environments that recreate realistic seeming but simulated hazardous situations. We propose VR as the next-generation tool to study RT processes, taking advantage of the big four families of metrics which can provide objective assessment methods with high ecological validity: the real-world risks approach (high presence VR environments triggering real-world reactions), embodied interactions (more natural interactions eliciting more natural behaviors), stealth assessment (unnoticed real-time assessments offering efficient behavioral metrics) and physiological real-time measurement (physiological signals avoiding subjective bias). Additionally, VR can provide an invaluable tool, after the assessment phase, to train in skills related to RT due to its transferability to real-world situations.

## Introduction

Each year, deficient Occupational Safety and Health (OSH) practices cause a global cost of approximately 2680 billion euros (Elsler et al., 2017). Although OSH training has shown positive impacts in the workplace, its effectiveness is below expectations (Robson et al., 2012). It has been demonstrated that the natural differences between individuals can appreciably influence this low effectiveness at several levels, cognitive, motivational and functional, among others (Motowildo et al., 1997). Risk propensity, defined as the “willingness to take risks” (MacCrimmon and Wehrung, 1990) and risk perception, defined as the individual’s assessment of how risky a situation is (Baird and Thomas, 1985), have been shown to have strong influence on risky decision making behaviors (Sitkin and Weingart, 1995). The measurement of risk taking (RT) attitudes is a recognized challenge for researchers and practitioners. Researchers have mostly employed self-report instruments to assess individual constructs based on theoretical psychological models (Brockhaus Sr, 1980; Ford et al., 1990; Gullone et al., 2000; Portell and Solé, 2001; Steinberg, 2004; Gardner and Steinberg, 2005; Sneddon et al., 2013; Rodríguez-Garzón et al., 2015). We have not found any one model that defines RT, thus its measurement requires further investigation. Lejuez et al. (2002) developed and validated a laboratory-based behavioral measure of RT (Balloon Analog Risk Task – BART). While this is a validated tool that has been used in several studies, we believe that it is desirable to develop a more ecological system to measure RT. VR provides the capability of creating interactive environments in which users can perform while their behavioral responses are recorded (Parsons, 2015). Accordingly, we propose that virtual environment based assessments are tools that can enhance the ecological validity of the evaluation of the responses evoked (Parsey and Schmitter-Edgecombe, 2013).

In this article we focus on the measurement of RT using physiological and behavioral metrics, with VR being employed as a tool to create immersive situations. We propose to use VR to assess RT attitudes under the paradigm of stealth assessment. VR can provide engaging virtual worlds which will allow real time measurement of RT behaviors.

This paper is comprised of four sections. In the first we review the theoretical framework of RT in the previous literature. In the second we summarize the extant instruments for the measurement of RT behaviors and discuss the current issues that make us believe that there is a need to establish a new approach. In the third we propose VR as a step forward in the assessment of RT. The fourth section briefly discusses the substantial implications raised by the article and our proposals for future research in this field.

## Research Into Risk Taking

RT research can be said to have started with the nuclear debate of the sixties. It was focused on risk acceptance and dealt with factors such as benefits and voluntariness. Since then, several more factors have been proposed for the explanation of RT: trust, trustworthiness and trust propensity (Colquitt et al., 2007); supportive supervision, job autonomy and communication quality (Parker et al., 2001); problem framing and outcome history (Sitkin and Weingart, 1995); expected utility (Kahneman and Tversky, 1986); genre (Byrnes et al., 1999) and boredom (Schroeter et al., 2014).

**FIGURE 1:**
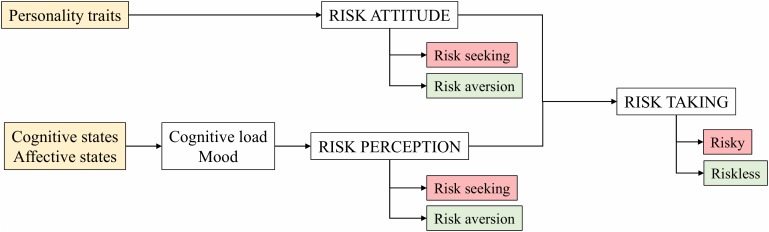
Individual differences that influence risk taking.

While these factors have been demonstrated to influence RT, individual differences constitute a key element in decision making processes (see Figure 1). According to Rundmo, 1996, a biased perception of risk – understood as the subjective evaluation of a risk - can lead to misjudgements of potentially hazardous risk sources. Therefore, if the subjective evaluation of a risk differs from the objective risk, this should be corrected (Risk Research Committee, 1980). Personality traits influence attitude toward risk, prompting risk seeking or risk aversion behaviors. This set of personal, innate, basic characteristics associated with risk were named Intrinsic Risk Attitude (IRA) by Schoemaker (1993) and have been shown to be consistent in various situations and contexts (Dohmen et al., 2011). Additionally, cognitive and affective states are also considered to be key influencers in the decision making process. We highlight mood and cognitive load as two main representative factors in this category. Mood has a strong influence on RT. People in a positive mood tend to focus on the benefits of a risky situation, much more so than those in neutral mood, making them more susceptible to undertake risky behaviors (Forgas, 1982, 1995; Forgas and Bower, 1987; Yuen and Lee, 2003). On the other hand, people in a negative mood overestimate risks and try to avoid potential loss and, therefore, think and act more carefully (Jorgensen, 1996). Cognitive load, the amount of mental activity involved in working memory, might also play a role in risk perception, since some kind of decisions, based on utilitarian judgments, require additional cognitive resources (Greene et al., 2008).

## Risk Taking Measures: Current Issues

RT measurement is a non-deterministic and non-standardized process based on different perspectives. Traditionally, most theories of human behavior are based on a model of the human mind that assumes that humans can think and verbalize accurately about their attitudes, emotions and behaviors (Simon, 1976; Brief, 1998). To date, most of the theoretical constructs used in RT assessment are based on explicit measures such as self-reports. However, recent advances in neuroscience have demonstrated that most of the brain processes that regulate our emotions, attitudes and behaviors are not conscious. That is, they are implicit processes that, in contrast to explicit processes, humans cannot verbalize (Barsade et al., 2009; George, 2009; Becker et al., 2011).

Several explicit measures of RT, oriented to evaluate attitude to risk, deferred risk perception or expected risk behavior, have been proposed in the last fifty years. Some authors have employed self-report measures based on questionnaires on compliance with safety practices in the workplace (Parker et al., 2001; Mohamed et al., 2009; Seo et al., 2015), attitude toward risk and organizational commitment (Kivimäki and Kalimo, 1993) and in studies into decision making (Sitkin and Weingart, 1995). On the other hand, some works have drawn on theoretical multidimensional models based on psychological constructs, such as personality (Lejuez et al., 2002; Skeel et al., 2007), impulsivity (Lejuez et al., 2002), sensation seeking (Horvath and Zuckerman, 1993; Lejuez et al., 2002) and situational awareness (Lejuez et al., 2002).

However, as in many other disciplines, pre- and post-experiment questionnaires have an important intrinsic bias since individuals’ cognitive and psychological states will be different when they answer the questionnaires to when they actually underwent the experiences that the researchers wish to analyse (Kivikangas et al., 2011). As stated in (Wang et al., 2015), this tendency is primarily due to “social desirability effects,” which can lead to untrue accounts of behavior, attitudes and beliefs (Paulhus, 1991). In addition, there may be different interpretations of specific self-report items, resulting in unreliability and poorer validity (Lanyon and Goodstein, 1997). Lastly, some self-reporting questions need people to possess overt knowledge of their dispositions (Schmitt, 1994) and this does not always run true.

To our knowledge, the BART (Lejuez et al., 2002) constitutes, to date, the only tool for RT measurement using implicit measures. The authors developed and validated a laboratory-based behavioral measure of risky behaviors. In this task, a balloon was presented in the middle of the screen. Subjects were asked to pump it as much as possible, knowing that it could exploit at any time. Participants were told that they would obtain a financial reward the more they could inflate the balloon without breaking it. Although the reliability of this tool has been retested (White et al., 2008), extensive investigations have demonstrated that the correspondence between performance in neuropsychological tests and real-life behaviors is very weak (Manchester et al., 2004; Sbordone, 2008; Bottari et al., 2009).

In the BART validation study, researchers employed measures of impulsivity, sensation seeking and behavioral constraint. We consider this a good basis to build on, since each of these constructs has been investigated independently and associated with RT. Firstly, impulsivity has been associated with RT in terms of drug use, drink driving and seatbelt use (de Wit, 2009; Stanford et al., 1996). Some authors have also demonstrated its connection with emotional self-control, inhibition and, especially, the management of frustrating situations (Cooper et al., 2000; Boyer, 2006). In addition, researchers have studied the relationship between the sensation seeking trait and RT in several domains, such as recreation, health, career, finance, safety and social life (Nicholson et al., 2005). Donohew et al. (1999) concluded that sensation seeking is an important factor in sexual RT. According to Tellegen’s (1985), model behavioral constraint is one of the dimensions that composes personality. The behavioral constraint factor encompasses control, harm avoidance and traditionalism facets. In the same way, there is empirical evidence of the influence of personality traits on RT attitudes, in particular punishment avoidance (Paulus et al., 2003). We can find an interesting study from Wills et al. (2006) supporting this idea in the substance abuse field.

## Limitations of Current Risk Taking Measures

As mentioned previously, to date the majority of RT assessment tools has been based on explicit measures and the use of questionnaires.

BART, with its multi-dimensional set of psycho-cognitive influences, represents the only alternative to explicit measures of RT behavior, but its design has some intrinsic limitations that current technologies could help to overcome.

In this regard, we believe that the existing measurement instruments do not reflect real situations, in which the subjects can perform as in real life, which leads to skewed results. In the laboratory the controlled stimuli given to subjects often do not include variables that are present in real life situations. Thus, the ecological validity of these methodologies, such as BART, is quite limited. Furthermore, these measurement tools do not involve any strong physical interaction, but require only simple actions, such as clicking a mouse, ignoring the influence of the reactions of the rest of the body. In addition, when an individual is submitted to the currently available tests, (s)he is aware that (s)he is being assessed and can alter the outcomes; so we propose stealth assessment as a means of obtaining reliable results about real behaviors unnoticed by the subject. Lastly, we suggest that physiological processes must be considered as important measures of RT, as these measurements are uncontaminated by the participant’s answering style, social desirability, interpretations of questionnaire item wording, the limits of his or her memory or by observer bias (Kivikangas et al., 2011). Thus, we propose an alternative measurement method which aims to advance in four specific aspects:

(1) Real-world risks: As stated in Bornovalova et al. (2009), p.261. “[BART] …… did not collect information on “real-world” risk-taking. It would be of both theoretical interest and clinical relevance to examine whether the current results “hold” when considering actual risk-taking behavior”. We want to expose individuals to (almost) real risks in order to obtain (almost) real reactions. Amit et al. (2014) found that humans demonstrate two kinds of thought processes in any given situation, verbal and visual. A person who tends to verbal thinking builds meanings using words. This generates an abstract interpretation of a concept. It is usual, in this circumstance, to exhibit controlled cognitive processes, experience high psychological distance and to make utilitarian judgements. In contrast, visual thinking is associated with the use of images to represent concepts, generating a sense of proximity and the making of deontological judgements. People who tend toward visual thinking are willing to be guided by emotional automatic processes and are strongly influenced by secondary emotions. Using the real-world risks approach, we suggest that we can evoke the sensation of physical risk and initiate visual thinking that would arise in a real life, risky situation.
(2) Embodied cognition: How the actions of our bodies influence our perception, communication and learning processes is a field of study known as Embodied Cognition (EC). EC can be defined by stating that cognition is solidly based on corporal interactions with the physical environment (Wilson, 2002; Gallagher, 2005). Going into more detail, systems for sensing, acting and thinking are intrinsically interdependent and human cognition is made up of complex, specific representations combining all three systems (Soler et al., 2017). During recent years, instructional methods based on bodily interactions have been developed to create meaningful connections between physical activity and different knowledge domains, mainly in the STEM (Science, Technology, Engineering and Maths) area, strongly linked to the new Mixed Reality media (Lindgren and Johnson-Glenberg, 2013). To a certain extent, embodied learning could represent an important foundation on which to build a whole set of interactive, immersive learning environments. This concept is supported by previous research (Kontra et al., 2012) that argues that taking a meaningful action enhances learning in comparison to passively perceiving that action. This idea has been strongly supported for decades by classical learning theorists such as Piaget and Cook (1952) and Vygotsky (1978). We propose to take advantage of the ideas underlying embodied learning theory and use high level cognitive experiences, involving sensing, acting and thinking, to measure and change attitudes in a deeper, more effective way.
(3) Stealth assessment: “When embedded assessments are seamlessly woven into the fabric of the learning environment so that they are virtually invisible or unnoticed by the learner, this is stealth assessment” (Shute and Spector, 2008, unpublished, p.2). More specifically, this method offers the possibility of assessing different behaviors related to concrete capabilities, providing indirect evaluations in real time (Mislevy et al., 2003) and reducing test anxiety, while maintaining validity and reliability (Shute et al., 2008). Stealth assessment fits into the framework of evidence-centered design (ECD), which considers three conceptual models that must be present in stimuli design: the competency model, which aims to define the skills that the researcher wishes to assess; the evidence model, that aims to define specific behaviors and their relationships with particular skills and capabilities; and the task model, which is designed to develop specific scenarios and tasks to prompt skills-related behaviors (Shute, 2011). Thus, stealth assessment allows the setting of tasks and creation of situations that can elicit particular behaviors connected with the skills and capabilities to be evaluated.
(4) Physiological real-time measurement: Several physiological measures have recently been proposed as implicit measures of human behavior (Kivikangas et al., 2011). Skin conductance level has been successfully used as a measure of implicit processes such as stress, affective arousal and cognitive processing (Sequeira et al., 2009). Heart variability (HV) has been used for the implicit measurement of complex phenomena, for example cognitive load (Durantin et al., 2014). Eye tracking (ET) is a very interesting measure of subconscious brain processes, showing correlations with information processing in risky decisions (Glöckner and Herbold, 2011) and problem solving (Knoblich et al., 2001). Recent studies, using Functional Near-Infrared Spectroscopy (fNIRS), into decision making under pressure (Tsujii and Watanabe, 2010) and decision making processes in approach-avoidance theories (Ernst et al., 2013), are highly relevant for RT measures.

## Virtual Reality and Risk Taking Assessment

Virtual Reality is a 3D synthetic environment able to simulate real experiences in which subjects can interact as if they were in the real world (Alcañiz et al., 2003). VR provides greater immersion, fidelity and higher level of active user involvement than traditional methods of assessment and training (Hedberg and Alexander, 1994). In our view, VR constitutes a suitable tool for behavioral measurement, since it complies with the requirements (see Table 1) of the four specific aspects discussed in the previous section: (1) the real-world risks approach, (2) embodied learning, (3) stealth assessment and (4) physiological real-time measurement.

**Table 1:** VRfeatures and benefits of risk taking measurement.

Domain	VR features	Benefits of measurement
Real-world risks	Evokes the sensation of physical risk	Neural mechanisms similar to real life
Embodied interactions	Actions raised in the first person	More emotional decisions
Stealth assessment	Indirect evaluation in real time	Reduction of test anxiety More validity and reliability
Physiological real-time measurement	Physiological measurement during performance	Involuntary, uncontaminated by participant answering bias


(1) According to Slater (2009), the result of immersion through technology is the psychological state of “being there,” where the subject essentially forgets that (s)he is in a virtual reality setting. This produces a sense of presence and a “plausibility illusion” which evoke the perception that what is happening in the VR is actual and allows subjects to interact and behave as they might in real life. VR is being used increasingly for natural phenomena and social interactions simulation, since it has been demonstrated that neural mechanisms in humans when they are immersed in a virtual environment are similar to those in real life (Alcañiz et al., 2009). When we talk about training and learning, failure is a necessary ingredient. There is evidence that people who have faced real hazards have a more cautious attitude toward OSH (Cavalcanti and Soares, 2012). Hazards in real life can involve serious danger. This is why VR emerges as a potential medium for RT assessment and training, allowing users to operate, without risks, in a quasi-real environment (Amokrane et al., 2008). VR allows the exposure of a person to a risky situation and the activation of high fidelity cognitive processes and behaviors due to the plausibility of the immersion. (2) VR environments allow users to take part in an embodied learning experience, mainly through physical interactions (Kilteni et al., 2012). Going further with this concept (Dourish, 1999, unpublished), we consider a virtual interaction to be fully embodied when it is believable, in the sense of using our body coherently as we do in the real world. The dual-process theory of moral judgment, when it refers to moral dilemmas, makes a distinction between personal and impersonal dilemmas (Greene et al., 2001; Greene, 2009): personal dilemmas are conflicts in which the subject experiences the situation in the first person and actions are carried out physically – e.g., pushing. Conversely, impersonal dilemmas are seen from the outside, and the subjects do not take overt physical actions, but make only minor responses, such as pressing switches or levers. Based on this distinction, it has been demonstrated that when actions are based on the first person perspective and involve physical acts, the subjects tend to make more emotional decisions (Greene et al., 2001; Amit et al., 2014). (3) Stealth assessment can be also defined as a performance-based method, in which what is evaluated is latent (Rupp et al., 2010). Under this paradigm, embedding assessments in immersive virtual worlds is an innovative approach (Shute and Spector, 2008) that, in our view, is an improvement from the standpoint of ecological validity. (4) Regarding physiological real-time measurement, VR provides interactive and multimodal sensorial stimuli that provide unique advantages over other methodologies in neuroscientific investigation (Bohil et al., 2011). Thus, due to technological advances, researchers can now use accurate, affordable devices to obtain physiological measures which have been found to be more effective than self-reported measures as they (a) are not intrusive, (b) do no rely on participants’ self-assessment of their emotional or cognitive experience, and (c) can detect changes in participants in real time. We have previous experience in combining VR technology with brain activity measures, and these results have shown that interactive virtual environments allow the measurement of emotional responses (Marín-Morales et al., 2018).

For these reasons, customizable, domain independent VR environments, in which individuals can, to a certain extent, act freely and react naturally to different risks or hazards, open to researchers an uncharted field of information about RT attitudes and behaviors. The set of these requirements may result in an application that includes a virtual environment, with a specific narrative that face the users with risky situations. This should be designed following stealth assessment methodology, and would allow physiological and behavioral measurement to provide information about individual decision making in the field of RT. We will show an example of how this tool might perform: the user could be in a virtual environment that consists in a path which (s)he must cover from start to finish, within the shortest possible time. Suddenly, (s)he meets a bifurcation, where (s)he has to choose whether a safe but log way – less risk, less potential benefit -, or a dangerous but short path – higher risk, higher potential benefit -. During this decision making process, we could take measures of galvanic skin response to assess emotional activation, and behavioral measures such as reaction time and the decision made by the user. As a result, we could obtain information about specific weight of emotional processes in RT, and its influence on behavior.

Our future research aims to study to what extent a VR tool is able to measure the cognitive and affective processes that influence RT. Furthermore, we would focus on how virtual interactions and narratives weight on the decision making process.

## Conclusion

RT measurement is a major challenge for companies and researchers. Investigations into behavioral measurement are at a turning point as, due to the potential of technological advances, we can generate virtual worlds to evaluate and, going further, train people in certain skills and competences. We suggest that virtual reality is the most appropriate medium for assessing attitudes to risk and risk perception, conditioning factors in the RT process, due to their immersive capabilities. We propose to undertake future investigations into real-world risks, embodied interactions, stealth assessment and physiological real-time measurement as differentiating elements in RT assessment. If we can study and measure the real, unbiased reactions of people facing risky or hazardous situations, it will be possible to create customized training programs to fit their individual characteristics. This can be expected to contribute to the improvement of OSH training programs, reducing work-related incidents and, consequently, costs for companies.

## Author Contributions

MA, CdJ, and NÁ were responsible for the general idea of the paper. CdJ and JS participated in drafting the work, while JG and MC revised it in-depth and provided new ideas thanks to their previous experience. MA supervised the entire work, revised the manuscript and approved the final version to be submitted. All authors made substantial contributions to the conception and development of the work.

## Conflict of Interest Statement

The authors declare that the research was conducted in the absence of any commercial or financial relationships that could be construed as a potential conflict of interest.
